# Astroglial Connexin 43 Deficiency Protects against LPS-Induced Neuroinflammation: A TSPO Brain µPET Study with [^18^F]FEPPA

**DOI:** 10.3390/cells9020389

**Published:** 2020-02-07

**Authors:** Nicolas Vignal, Anne-Cécile Boulay, Carine San, Martine Cohen-Salmon, Nathalie Rizzo-Padoin, Laure Sarda-Mantel, Xavier Declèves, Salvatore Cisternino, Benoît Hosten

**Affiliations:** 1 Unité Claude Kellershohn, Institut de Recherche Saint-Louis, Faculté de Santé, Université de Paris, 75010 Paris, France; nicolas.vignal@aphp.fr (N.V.); nathalie.rizzo-padoin@aphp.fr (N.R.-P.); laure.sarda-mantel@aphp.fr (L.S.-M.); 2 Therapeutic Optimisation in Neuropsychopharmacology, INSERM UMR-S 1144, 75005 Paris, France; xavier.decleves@aphp.fr (X.D.); salvatore.cisternino@aphp.fr (S.C.); 3 Hôpital Lariboisière: Service de médecine nucléaire, Assistance Publique–Hôpitaux de Paris, 75010 Paris, France; 4 Center for Interdisciplinary Research in Biology (CIRB), College de France, CNRS, INSERM, PSL Research University, 75005 Paris, France; anne-cecile.boulay@college-de-france.fr (A.-C.B.); martine.cohen-salmon@college-de-france.fr (M.C.-S.); 5 Hôpital Saint-Louis: Service Pharmacie, Assistance Publique–Hôpitaux de Paris, Unité Claude Kellershohn, 75010 Paris, France; carine.san@aphp.fr; 6 Faculté de Santé, Université de Paris, 75005 Paris, France; 7 Hôpital Cochin: Service de biologie du médicament et de toxicologie, Assistance Publique–Hôpitaux de Paris, 75014 Paris, France; 8 Hôpital Necker–Enfants Malades: Service de pharmacie, Assistance Publique–Hôpitaux de Paris, 75015 Paris, France

**Keywords:** [^18^F]FEPPA, astrocytes, autoimmunity, blood–brain barrier, brain inflammation, Connexin 43, PET imaging, TSPO

## Abstract

Astroglial connexin 43 (Cx43) has been recognized as a crucial immunoregulating factor in the brain. Its inactivation leads to a continuous immune recruitment, cytokine expression modification and a specific humoral autoimmune response against the astrocytic extracellular matrix but without brain lesions or cell lysis. To assess the impact of Cx43 deletion on the brain’s inflammatory response, TSPO expression was studied by positron emission tomography (PET) imaging with a specific radioligand, [^18^F]FEPPA, in basal conditions or upon Lipopolysaccharides (LPS)-induced inflammatory challenge. Astroglial Cx43-deleted mice underwent [^18^F]FEPPA PET/CT dynamic imaging with or without LPS injection (5 mg/kg) 24 h before imaging. Quantification and pharmacokinetic data modelling with a 2TCM-1K compartment model were performed. After collecting the mice brains, TSPO expression was quantified and localized by Western blot and FISH analysis. We found that astroglial Cx43 deficiency does not significantly alter TSPO expression in the basal state as observed with [^18^F]FEPPA PET imaging, FISH and Western blot analysis. However, deletion of astrocyte Cx43 abolishes the LPS-induced TSPO increase. Autoimmune encephalopathy observed in astroglial Cx43-deleted mice does not involve TSPO overexpression. Consistent with previous studies showing a unique inflammatory status in the absence of astrocyte Cx43, we show that a deficient expression of astrocytic Cx43 protects the animals from LPS-induced neuroinflammation as addressed by TSPO expression.

## 1. Introduction

There is a growing body of evidence that brain inflammation and immune responses are involved in the pathogenesis of some psychiatric diseases [1,2,3]. For example, in autoimmune encephalitis (e.g., anti-NMDA receptor encephalitis), autoantibodies are unable to activate the lytic complement system pathway which may lead to psychiatric disorders [2,4]. Brain inflammatory status can be assessed with noninvasive imaging methods such as positron emission tomography (PET) using a radioligand targeting de novo or overexpressed proteins/biomarkers of neuroinflammation. The 18 kDa translocator protein (TSPO) is known to be at least a hallmark of microglial activation [5]. TSPO PET imaging using specific radioligands, such as [^18^F]FEPPA, was successfully used to assess brain inflammation in neurological diseases (e.g., Alzheimer [6], Parkinson’s disease [7]) and in autoimmune diseases (e.g., multiple sclerosis [8]). However, the inflammatory status assessed by TSPO PET imaging remains controversial in depression [9] and psychosis [10]. The mechanisms behind the potential correlation between inflammation, immunity and psychiatric diseases need to be further explored. Thus, new preclinical models are required for addressing this issue.

Connexin 43 (Cx43) is a gap junction protein, highly expressed in brain astrocytes. Several studies have shown that Cx43 is involved in the cognitive functions and behavior in mice and its expression can change in several psychiatric conditions [11,12,13,14]. Interestingly, Cx43 is a key immunoregulating brain factor [11]. Indeed, prior studies have demonstrated that in mice, a deficiency of astroglial Cx43 leads to a transient activation of brain capillary endothelial cells, a continuous immune recruitment, and the development of a specific humoral autoimmune response without complement system activation and obvious behavioral symptoms [15]. The deletion of astrocytic Cx43 also alters the expression of cytokines and chemokines in the brain, with an increase in pro-inflammatory cytokines such as IL-1β, TNF-α and chemokines (e.g., Ccl5), but also an increase in anti-inflammatory cytokines such as IL-10 [16]. Indeed, brain recruited lymphocytes express IL-10 and IFN-γ in 3-month-old KO Cx43 brain mice, suggesting a pro-inflammatory as well as an anti-inflammatory status [15]. LPS is a compound known for its efficiency in inducing systemic inflammation disease in mice, including neuroinflammation with microglial activation [17]. Focusing on the widely studied TSPO protein, the aim of this work was to investigate the neuroinflammatory status in these mice before and after LPS challenge, by using TSPO PET imaging as a marker of microglial activation.

## 2. Materials and Methods

### 2.1. Animals

Three-month-old female and male mice were used in this study and kept in pathogen-free conditions. Astrocyte-targeted Cx43 knockout (KO) (Cx43^fl/fl^/hGFAP-Cre = KO Cx43) and floxed (FL) mice (Cx43^fl/fl^ = FL Cx43) were provided by the CIRB animal facility (Collège de France, Paris) [15]. The Cre-recombinase activity in the brain [18] was tested systematically by assessing beta-galactosidase activity (Roche^®^, Meylan, France). To induce brain inflammation, 5 mg/kg of LPS from *Salmonella enterica serotype typhimurium* (Sigma-Aldrich^®^, Saint Quentin-Fallavier, France) or 500 µL of saline was injected intraperitoneally into mice 24 h before [^18^F]FEPPA-PET/CT imaging. All animal experiments were performed in accordance with the European Guidelines for Care of Laboratory Animals (2010/63/EU) and were approved by the Animal Ethics Committee of Paris Nord (APAFIS#2768-20l5l11314249747).

### 2.2. Reagents for Radiochemistry

All reagents and solvents were purchased from commercial suppliers (ABX^®^, Radeberg, Germany or Sigma-Aldrich^®^) and were used without further purification.

### 2.3. [^18^F]FEPPA Radiosynthesis and PET/CT Imaging

[^18^F]FEPPA radiosynthesis and control quality were performed as previously described [19]. [^18^F]FEPPA radiochemical purity was more than 99% and its molar activity at the end of synthesis was 183 ± 80 GBq/µmol. During radiotracer administration and image acquisition, mice were anesthetized with 2.5% and 1–1.5% isoflurane in oxygen at 0.8–1.5 L/min and 0.4–0.8 L/min respectively for induction and maintenance. PET/CT studies investigating brain inflammation were performed after the injection of [^18^F]FEPPA diluted in 150 µL saline (10 MBq) into the lateral tail vein of mice. The injection was made on an Inveon micro PET/CT animal scanner (Siemens Medical Solutions^®^, Saint-Denis, France) with a spatial resolution of 1.4 mm full width at half-maximum at the center of the field of view. Dynamic mod-list PET acquisitions of the whole-body mice were performed from the time of the radiotracer injection until 60 min after the injection (n = 12 FL and 12 KO Cx43) and followed by a 3-min duration CT acquisition. PET data were reconstructed using 3-dimensional ordered-subset expectations maximization algorithm into a 128 × 128 image matrix (21 frames: 3 × 5, 3 × 15, 4 × 30, 3 × 60, 2 × 120, 4 × 300, 2 × 900 s and were corrected for random, scatter and decay events.

### 2.4. Image Analysis and Pharmacokinetic Modeling

**Figure 1:**
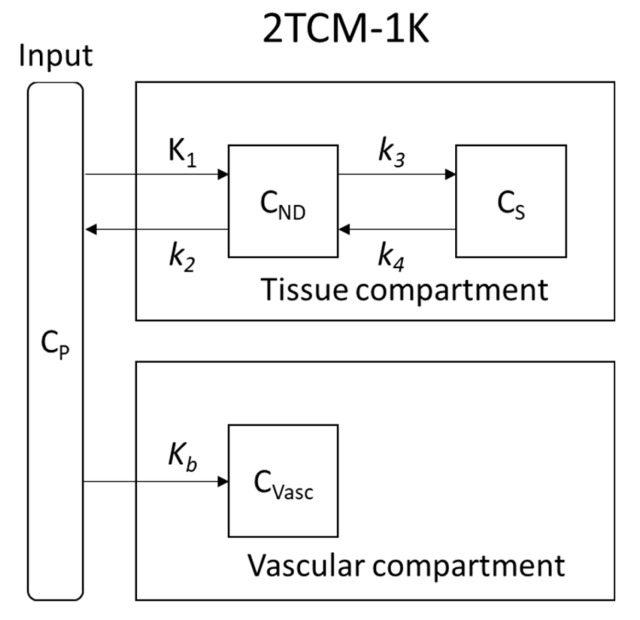
2TCM-1K pharmacokinetic model: K_1_ and k_2_ are the rate constant between the plasmatic compartment (C_P_) and the non-displaceable compartment (C_ND_, free and non-specific fixation); k_3_ and k_4_ are the rate constant for input and output, respectively, between C_ND_ and specific fixation compartment (C_S_). K_b_ is the input rate constant between C_P_ and the vascular non-reversible fixation compartment (C_VASC_).

PET/CT images were visually assessed and quantified using PMOD^®^ version 3.806 image analysis software (PMOD Technologies^®^, Zurich, Switzerland). For comparisons, all values of radioactivity concentrations were normalized by the injected dose and expressed as a percentage of the injected dose per g of tissue (% ID/g). To achieve a more reproducible method, an automatic mode of regions of interest (ROI) drawing was used. Automatic rigid matching was applied to PET images with their corresponding CT. Then, the two matched images were cropped so as to isolate the brain area. The cropped and matched CT image was automatically rigid matched with a predefined T2 MRI mouse brain atlas template (M. Mirrione, included in PMOD). The transformation of the CT image was then applied to the corresponding PET image. Once the ROI drawing was completed, the time activity curve (TAC) of each brain region was obtained. Only the whole brain, the cortex and the hippocampus were studied due to the small volume of each mouse brain. The arterial input function was computed from samples of plasma and corrected for the metabolism of the parent ligand as we previously described [19]. A vascular trapping 4 rate-constant kinetic (2TCM-1K) model with two compartments (Figure 1) was used to characterize [^18^F]FEPPA pharmacokinetics [20,21,22].

### 2.5. Western Blot

After imaging, mice were sacrificed and their brain regions (cortex and hippocampus) dissected. Samples were reduced in powder at −80 °C and immediately dissolved in PBS with 2% SDS and 1× EDTA-free Complete Protease Inhibitor (Roche^®^). The lysates were sonicated twice at 10 Hz (Vibra cell VCX130) and centrifuged for 30 min at 16,000× *g* at 4 °C. Supernatants were boiled in Laemmli loading buffer. Protein content was measured using the BCA protein assay reagent (Thermo Fisher^®^, Les Ulis, France). Equal amounts of proteins (20 µg) were separated by denaturing electrophoresis in NuPAGE^®^ 4–12% Bis-Tris acetate gradient gel (Thermo Fisher^®^) and electro-transferred to nitrocellulose membranes. The membranes were analyzed using the following primary antibodies: rabbit anti-TSPO (Abcam) (1:10,000); horseradish-peroxidase-conjugated (HRP) anti-GAPDH (1:10,000); and the secondary antibodies, HRP-conjugated anti-rabbit (GE Healthcare) (dilution 1:2000). HRP activity was visualized by enhanced chemo-luminescence (ECL) using Western Lightning plus an enhanced chemoluminescence system (Perkin Elmer^®^, Waltham, MA, USA). Chemoluminescence imaging was performed on a LAS4000 (Fujifilm^®^,Tokyo Japan). GAPDH expression was used as a loading reference.

### 2.6. Fluorescent in Situ Hybridization

The complete protocol is available in [23], describing an adaptation of the RNAScope^®^ procedure (Advanced Cell Diagnostics Inc., Hayward, CA, USA). Briefly, 4% PFA-fixed 40 µm-thick brain slices were treated for 10 min with RNAscope^®^ hydrogen peroxide solution, mounted on Super Frost plus^®^-treated glass slides. The unmasking of mRNAs was achieved by a 15-min incubation at 100 °C with RNAscope^®^ Target Retrieval Reagent and a 30-min treatment at 40 °C with RNAscope^®^ Protease-plus. Fluorescent in situ hybridization (FISH) was then performed using the RNAscope^®^ Multiplex Fluorescent Reagent Kit v2 (Advanced Cell Diagnostics Inc., Hayward, CA, USA), according to the manufacturer’s instructions, with a TSPO probe and an Opal 570 (FP14488A, Perkin Elmer^®^, Waltham, MA, USA).

The images were acquired using a Leica^®^ SP5 confocal microscope (Leica microsystems Gmbh, Wetzlar, Germany) with a 40× objective and were analyzed using FIJI software (v1.52p). FISH labelling was segmented via a Gaussian Blur (Sigma = 1), background substraction (rolling ball radius = 5 pixel) triangle threshold. Particles of 1–10 µm size were selected for FISH quantification.

### 2.7. Statistical Analysis

Data are presented as mean ± SD unless indicated otherwise. A statistical analysis was performed using Prism 5^®^, version 5.0.1. A significance value of *p* < 0.05 was used in a Student (*t*-test), a Mann-Whitney (U-test) or a one-way ANOVA test.

## 3. Results

### 3.1. Astrocyte Cx43 Deletion Does Not Alter [^18^F]FEPPA Brain Distribution

**Figure 2:**
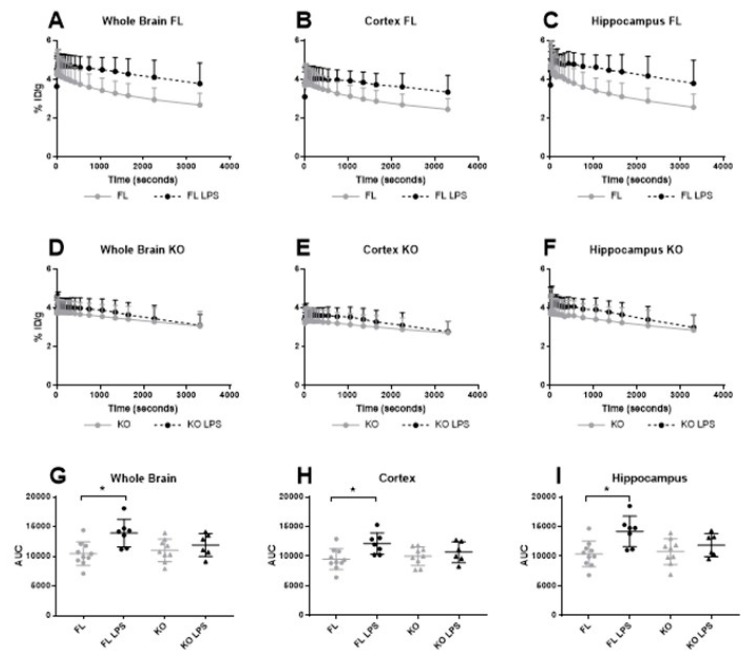
Time activity curve and the area under curve (AUC) of [^18^F]FEPPA in the whole brain (**A**,**D**,**G**), the cortex (**B**,**E**,**H**) and in the hippocampus (**C**,**F**,**I**) in floxed and deleted Cx43 mice with or without LPS injection 24 h before imaging. [^18^F]FEPPA Time Activity Curves (TAC) (in %Injected Dose/g) and the AUC (%ID×g^−1^ × s^−1^) before and after LPS injection in FL and KO Cx43 mice (n = 6–10). (**A**,**D**,**G**) [^18^F]FEPPA in the whole brain before and after LPS injection in FL and KO mice. (**B**,**E**,**G**) [^18^F]FEPPA in the cortex before and after LPS injection in FL and KO mice. (**C**,**F**,**I**) [^18^F]FEPPA in the hippocampus before and after LPS injection in FL and KO mice. A one-way ANOVA test has been used for comparison between groups. * *p* < 0.05.

The inflammatory statuses of the brains were assessed in 3-month-old FL and KO Cx43 mice with [^18^F]FEPPA PET imaging. [^18^F]FEPPA regional TAC for the whole brain, the cortex and the hippocampus showed no significant difference in [^18^F]FEPPA uptake between KO (n = 7) and FL Cx43 (n = 7) groups (Figure 2A–F). Accordingly, the area under curve (AUC) of the [^18^F]FEPPA activity in the three regions showed similar results (Figure 2G–I). Astrocytic Cx43 deletion did not alter [^18^F]FEPPA distribution in 3-month-old mice.

**Figure 3:**
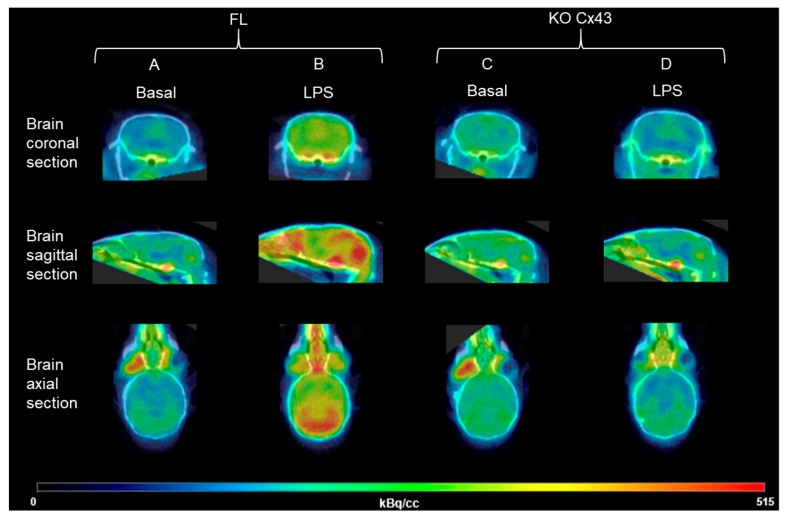
Representative microPET/CT imaging of brain inflammation with the TSPO radioligand [^18^F]FEPPA before and after the LPS injection in FL or KO Cx43 mice. The signal sum of all the frames in kBq/cc; (**A**) KO Cx43 mouse before the LPS injection, (**B**) KO Cx43 mouse after the LPS injection, (**C**) FL mouse before the LPS injection, (**D**) FL mouse after the LPS injection

To study the impact of astrocytic Cx43 deletion on LPS-induced neuroinflammation, [^18^F]FEPPA biodistribution was determined in FL (n = 5) and KO Cx43 (n = 5) mice before LPS i.p. injection and 24 h after the injection. [^18^F]FEPPA cerebral distribution was significantly higher after LPS injection in FL Cx43 mice, in the whole brain as well as in the cortex and the hippocampus (Figure 2A–C). The same results were observed regarding [^18^F]FEPPA AUC, with a statistically significant increase after the LPS injection (*p* = 0.0119, *p* = 0.0276 and *p* = 0.00930 in the whole brain, the cortex and the hippocampus, respectively) (Figure 2G–I). The increase in [^18^F]FEPPA concentration (in kBq/cc) is illustrated in Figure 3A,B. The injection of LPS did not change the [^18^F]FEPPA cerebral distribution in KO Cx43 mice (Figure 2D–F and Figure 3C,D).

**Figure 4:**
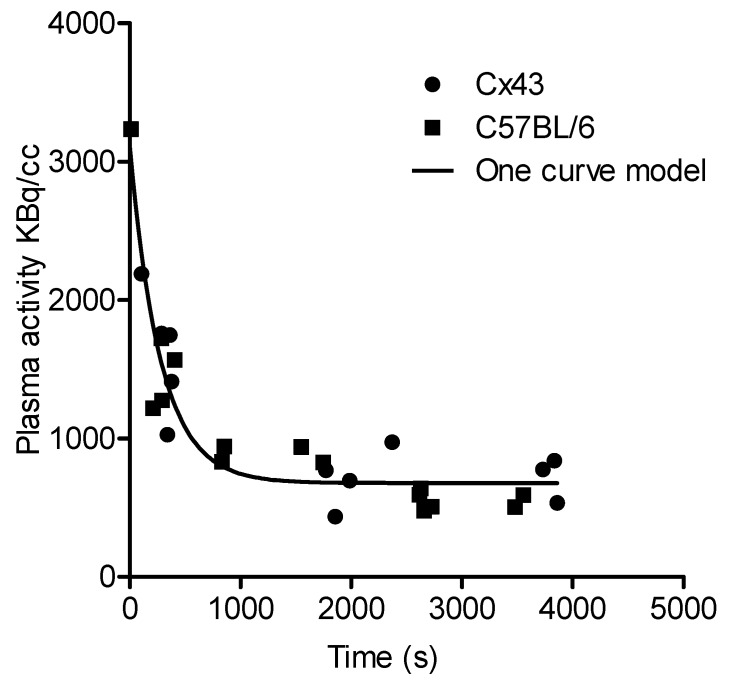
[^18^F]FEPPA arterial input function from Cx43 and C57BL/6 mice and the one curve model for all data sets.

A 2TCM-1K model was used for pharmacokinetic modelling to further evaluate [^18^F]FEPPA brain uptake. The [^18^F]FEPPA arterial input function from FL and KO Cx43 mice was computed and compared to the data previously obtained from C57BL/6 mice [19] (Figure 4). A statistical analysis revealed no differences between the two arterial input functions (*p* = 0.6899, Fisher exact test).

The one curve model best fit all the data and was used for pharmacokinetic modelling. In the 2TCM-1K model, the most relevant parameter to assert a modification of [^18^F]FEPPA cerebral distribution is the distribution volume *V*_T_ which showed a significant increase in FL Cx43 mice after the LPS injection (t-test mice, with a standard error > 50% for at least one parameter, were excluded, *p* < 0.05 [*]) but not in KO Cx43 mice (Table 1).

### 3.2. TSPO Brain Expression in FL Cx43 and KO Cx43 Mice with or without LPS Injection

**Figure 5:**
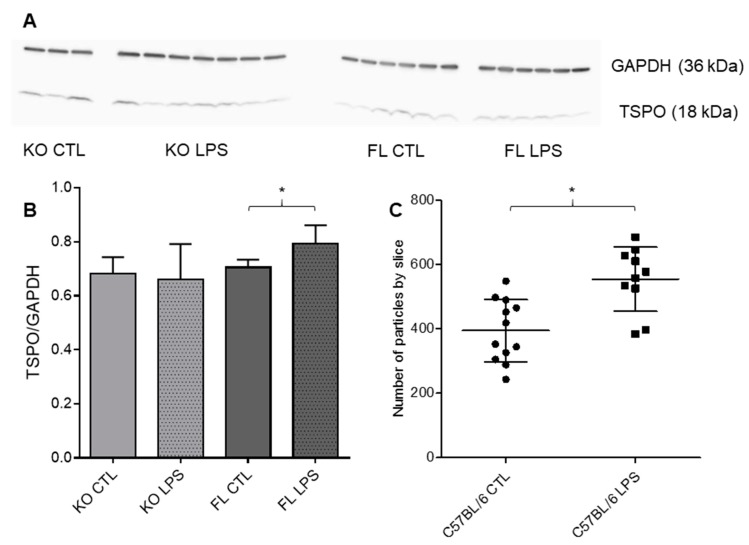
Effect of LPS injection on brain cortex TSPO expression (**A**) Blot of TSPO and GAPDH in the cortex of FL and KO Cx43 mice with or without the LPS injection. (**B**) Western blot analysis by mean comparison of TSPO/GAPDH ratio, significant difference between FL with or without LPS (*p* = 0.0276, t-test, n = 3–7). (**C**) FISH analysis by mean comparison of the number of particles in each cortex slice corresponding to a TSPO signal (*p* = 0.0011, t-test, n = 10–12). * *p* < 0.05.

To confirm, at the protein level, the observed discrepancies in LPS-induced [^18^F]FEPPA brain distribution between FL and KO Cx43 mice, we measured TSPO expression by Western blot (Figure 5A,B). Consistent with our PET results in basal conditions, no difference in TSPO expression was observed between FL and KO mice after saline injection. In fact, TSPO expression in the cortex was significantly increased after LPS injection in FL Cx43 mice (t-test, *p* = 0.0276). To confirm the increase in TSPO expression and to determine if the increase is translation- or transcription-dependent, we quantified TSPO mRNA by FISH. Similarly to the WB results, FISH signal in the C57BL/6 cortex after the injection of LPS was increased as compared to the control condition (Figure 5C).

Conversely, as we observed in our PET results, no increase in TSPO expression was observed by Western blot in KO Cx43 mice after LPS injection.

## 4. Discussion

In this preclinical study, we showed by [^18^F]FEPPA PET/CT imaging and confirmed by Western blot that TSPO brain expression, which reflects microglial activation, is similar in FL and KO Cx43 mice and increases after LPS administration in FL Cx43 mice but not in KO Cx43 mice.

While most of its functions remain unknown, Cx43 is a predominant protein expressed in astrocytes end-feet which is involved in many functions such as synaptic transmission, endothelial activation or immune reactions [24]. A previous study showed that 3-month-old astrocytic Cx43 deficient mice displayed an altered immune balance which includes an increase in pro-inflammatory and anti-inflammatory cytokines, leukocyte infiltration and an autoimmune response characterized by the presence of autoantibodies [11]. Therefore, the unique astrocyte profile and the mixed pro- and anti-inflammatory environment in those astrocytic Cx43 deficient mice could lead to a different response towards inflammatory stimuli from that observed in mice with normal expression of astrocytic Cx43. LPS peripheral injection induces neuroinflammatory reactions involving astrocytes [22]. It was previously reported that an acute i.p. injection of LPS leads to an increased brain expression of TSPO and a higher cerebral distribution of [^18^F]FEPPA [19].

In this study, we used a TSPO PET radioligand [^18^F]FEPPA to evaluate the potential impact of astrocytic Cx43 deletion on the brain inflammation process before and after LPS challenge. Before LPS challenge, we observed no significant difference in [^18^F]FEPPA cerebral distribution between KO Cx43 and FL Cx43 mice. Western blot analysis, performed after the imaging protocol, confirmed this result by showing a similar TSPO expression in the cortex of the two basal-state groups (Figure 5A,B). These results suggest that the altered immune balance in the KO Cx43 mice does not involve TSPO overexpression.

After LPS challenge, [^18^F]FEPPA distribution and TSPO expression in the brain of FL Cx43 mice were increased as previously seen in our work with C57BL/6 mice [19] and was confirmed by FISH analysis of TSPO RNA expression (Figure 5C). Conversely, there was no TSPO significant increase of [^18^F]FEPPA distribution and TSPO expression in the brains of KO Cx43 mice after LPS administration. To compare [^18^F]FEPPA brain distribution, pharmacokinetic modelling had to be performed due to the lack of reference tissue for the TSPO radioligands [20]. Brain pharmacokinetic modelling only showed a significant increase in *V*_T_ in FL Cx43 mice after LPS injection. No other statistically significant changes in constant rates between the plasma, the brain and vascular compartments were observed (Table 1).

*K*_1_ and *K*_b_, which represent, respectively, the uptake rate constant for compartment of non-displaceable tracer and the rate of binding to the endothelium of the vasculature, were not modified between FL and KO Cx43 ± LPS mice. This means that the four groups have comparable [^18^F]FEPPA passage across the blood–brain barrier [20,21]. This suggests that the significant increase in [^18^F]FEPPA *V*_T_ observed in FL Cx43 mice after LPS injection is more likely due to an increase in the brains’ parenchyma TSPO expression. In all groups, we observed a high variability in *k*_3_/*k*_4_ ratio, which is the ratio at the equilibrium of the specifically bound radioligand to the non-displaceable radioligand in the tissue. This variability is consistent with the relatively high standard deviation previously observed in [^18^F]FEPPA TAC (Figure 2). Additionally, Rusjan et al. have reported that the *k*_3_/*k*_4_ ratio is highly variable, and the most reliable parameter to document an increase in [^18^F]FEPPA brain distribution is the increase in *V*_T_ [25]. Furthermore, the same team has reported, by Monte-Carlo simulation, that an increase or decrease in *K*_1_ does not significantly modify *V*_T_ [25]. These results suggest that Cx43 astrocytic mice deficiency impairs the immune reactivity to LPS.

These results tend to show that astrocytic Cx43 plays a role in the brain immune balance. Indeed, deficient expression of Cx43 appears to decrease the inflammatory response to a peripheral LPS-induced inflammation. This hypothesis is in agreement with another study reporting the relationship between partial deletion of Cx43 in astrocytes and a weaker inflammation response upon LPS challenge using a neonatal sepsis mouse model [26]. Our study suggests that chronic immune recruitment observed in KO Cx43 mice may allow the brain to resolve inflammation more efficiently and to become more resilient to neuroinflammatory induction processes. Moreover, a recent transcriptomic study of KO Cx43 astrocytes has demonstrated that a quarter of the signalization pathways altered in this model involve inflammatory processes: chemokines involved in the immune recruitment such as Ccl5 and CxCl10 and anti-inflammatory markers such as IL-10 and CD274 (i.e., PD-L1, which inhibits lymphocyte T proliferation) are upregulated, whereas TLR4, for which LPS is a natural agonist, is downregulated [16]. Thus, KO Cx43 astrocytes seem to exist in a mixed inflammatory condition, which could impair their response to an LPS challenge.

Finally, other studies have reported a lack of changes to TSPO PET imaging in other models of neuroinflammation induction. For example, one imaging study with TSPO PET radioligand [^18^F]DPA-714 was unable to monitor the inflammatory process in a rat model of chronic morphine administration, even though brain inflammation was confirmed by an increase in pro-inflammatory cytokines [27]. These results hint that inflammatory processes may not always involve TSPO overexpression. Inflammatory phenomena seen in the model of Cx43 astrocytic deficiency seem to be similar to those observed in psychotic patients displaying encephalitis with autoantibodies against CNS antigens [2]. For a better understanding of the pathophysiology of autoimmune psychiatric diseases, it may be useful to consider further studies using the KO Cx43 model with alternative targets to assess neuroinflammation by non-invasive PET imaging.

## 5. Conclusions

This dynamic PET imaging study using TSPO radioligand [^18^F]FEPPA highlights the difference in neuroinflammatory reactivity in an autoimmune mouse model, characterized by a Cx43 inactivation of astrocytes, challenged with LPS, as compared to non Cx43 deficient mice. It also suggests that the altered immune balance in this model does not involve a TSPO expression modification. Further studies are necessary in order to better characterize inflammatory processes in this model.

## Figures and Tables

**Table 1:** Pharmacokinetics parameters for [^18^F]FEPPA distribution in the whole brain of FL Cx43 and KO Cx43 ± LPS groups (n = 5–7). Mean ± SD.

Group	*K*_1_ (mL × cm^−3^ × min^−1^)	*k*_2_ (min^−1^)	*k*_3_/k_4_	*K*_b_ (min^−1^)	*V*_T_ (mL × cm^−3^)
FL Cx43	1.31 ± 0.43	0.70 ± 0.34	0.65 ± 0.35	0.77 ± 0.12	3.24 ± 0.67
FL Cx43 + LPS	1.70 ± 0.44	0.70 ± 0.23	0.35 ± 0.24	1.11 ± 0.59	4.34 ± 0.68
*p* value	ns	ns	ns	ns	0.0204 *
KO Cx43	1.31 ± 0.48	0.75 ± 0.39	0.25 ± 0.33	0.99 ± 0.23	3.18 ± 0.63
KO Cx43 + LPS	1.32 ± 0.19	0.60 ± 0.25	0.19 ± 0.18	0.96 ± 0.22	3.95 ± 1.14
*p* value	ns	ns	ns	ns	ns

ns: non-significant, * *p* < 0.05.
